# Cervical Myelocystocele: A One-in-50,000 Congenital Abnormality of the Spinal Cord

**DOI:** 10.7759/cureus.37278

**Published:** 2023-04-08

**Authors:** Ali Msheik, Daniel Abbass, Mohamad Bayram, Ahmad Awde, Zeinab Al Mokdad

**Affiliations:** 1 Neurological Surgery, Zahraa Hospital University Medical Center, Beirut, LBN; 2 Neurological Surgery, Lebanese University Faculty of Medical Sciences, Hadath, LBN; 3 Neurological Surgery, Al Rassoul Al-Aazam Hospital, Beirut, LBN; 4 Neurosurgery, Endovascular Neurosurgery, Al Rassoul Al-Aazam Hospital, Beirut, LBN; 5 Medical Sciences, Public Health, Lebanese University Faculty of Medical Sciences, Hadath, LBN

**Keywords:** dysraphism, spina bifida, bowel dysfunction, bladder dysfunction, tethered spinal cord, myelomeningocele

## Abstract

Neural tube defects are a group of birth defects that affect the development of the spinal cord and brain. Myelomeningocele is a type of neural tube defect that results in the protrusion of the spinal cord and meninges through a defect in the vertebral column. While myelomeningocele is a relatively rare condition, cervical myelomeningocele is extremely uncommon. The condition can lead to various neurological problems and atrophies and is typically diagnosed in the first trimester of pregnancy using an ultrasound examination. Surgical intervention is typically recommended to repair the affected vertebral column. In this report, we describe the case of a four-month-old baby boy who was diagnosed with a cervical myelocystocele and successfully treated surgically. The patient had an excellent postoperative status, and this case highlights the importance of early diagnosis and intervention in the management of this rare condition.

## Introduction

Neural tube defects are a type of birth defect that include cranial conditions and open or firm spinal dysraphism [1]. They are second only to cardiovascular abnormalities in causing congenital morbidity and mortality [1,2]. Usually, these conditions are asymptomatic and do not require therapy. Out of 140,000 annual cases of neural tube defects, spina bifida presents around four to five per 10,000 births, with higher prevalence in whites vs. blacks and females vs. males [2,3]. Only 1,500 babies are born annually in the US with myelomeningocele, of which the cervical subtype is extremely rare (1-5% of all neural tube defects) [4]. An analysis in North China states that there is a 1.7% risk of a neural tube defect in pregnancy at an older age. A second study reports a 2%-3% recurrence of myelomeningocele in later pregnancies for women who had children with this birth defect [5].

At the primary phase of gastrulation, the neural plate is formed, which unfolds when the endoderm and the ectoderm complete the embryonic bilaminar circle and are then displaced, forming the mesoderm. Finally, the notochord with the ectoderm organizes the neuroectoderm. Spina bifida is a neural tube defect that occurs when the spinal column doesn't close properly during fetal development. It can present in different types, including spina bifida occulta, which is often asymptomatic, and myelomeningocele, which is the most severe form. If only the meninges herniate, it is called spina bifida cystica, while neural tissue devoid of any meningeal or skin covering is myeloschisis. Failure of the rostral neuropore to close leads to anencephaly [6].

Myelomeningioceles cause different neurological problems and various atrophies. Neurogenic bladder in children results from myelomeningocele. Patients may have paraplegia, sphincter disease, and sexual abnormalities with cardiotonic, nephritic, and pulmonary complications, reducing the survival rate [4,7]. Childhood symptoms include hypertonia, pain, and spinal malformations [7]. Multiple factors, including maternal (lack of folic acid, smoking), hereditary (chromosomal malformation), and environmental (contamination) factors, are correlated with myelomeningocele. Periconceptional folic acid supplementation prevents 50%-70% of spina bifida cases [8].

Myelomeningocele is identified in the first trimester of gestation. For at-risk patients, amniocentesis is recommended [2]. Ultrasound is generally adopted for second-trimester malformations such as hydrocephalus and microcephaly [2]. A fetal MRI can be useful in cases of an unclear diagnosis. The use of alpha-fetoprotein levels is advocated in screening. However, it is not a cost-effective approach [9].

Surgical intervention of the affected vertebral column is recommended [10]. Prenatal surgery is better to reduce aggravations in the future. Adequate sterilizing procedures, antibiotics, and routine radiological imaging are indispensable measures to mitigate infections and control the evolution of the patient's status. Late diagnosis of myelomeningocele leads to a higher mortality rate and more serious complications that include hydromyelia, muscle spasms, scoliosis, depression, suicide, obesity, and an allergy to latex [11,12].

This report presents the case of a four-month-old male baby boy who was referred to the neurological surgery department for a posterior cervical mass that was ultimately treated surgically and diagnosed as a cervical myelocystocele.

## Case presentation

Our patient was a four-month-old baby boy born with a small posterior cervical mass. The volume of the cervical mass kept increasing. The baby was playful and showed normal growth for his age with normal upper and lower limb, urinary, and gastrointestinal activity. The parents were concerned about the increase in the volume of the mass. No previous prenatal or postnatal examination was made. The baby was born in a rural hospital, and the parents refused medical intervention at his birth.

**Figure 1:**
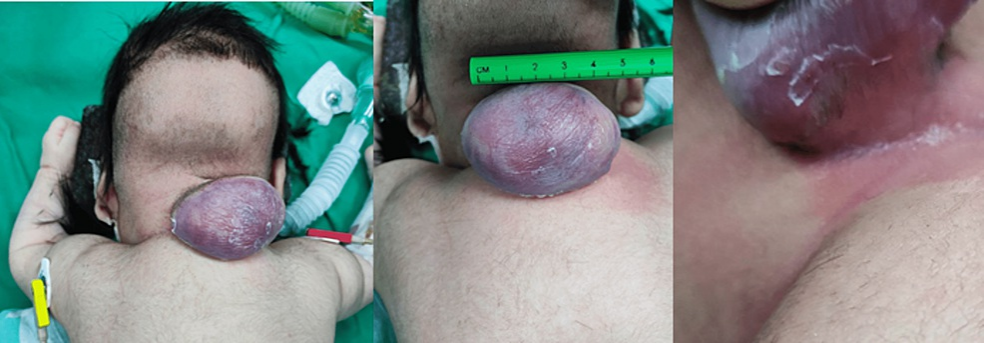
Preoperative images showing the shape of the cervical mass.

Physical examination revealed a pedunculated, egg-shaped mass connected by a stalk on top of the posterior cervical midline. The mass was enveloped by friable, purple-colored skin. The mass was compressible, moveable, warm to the touch, and translucent (Figure 1). The patient did not exhibit any signs of pain or jerks while the mass was being examined.

**Figure 2:**
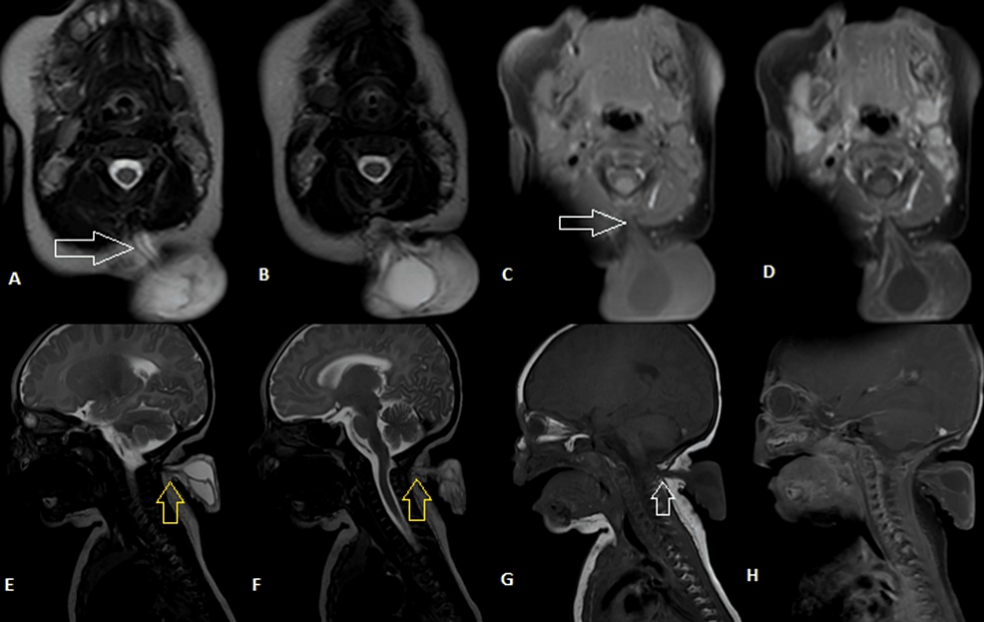
Cranial and cervical MRI of the patient after arrival at the outpatient clinic. A, B: axial T2-weighted images; C, D: axial T1-weighted images without and with gadolinium, respectively. The white arrow points to the connection between the cervical mass and the spinal cord; E, F: coronal T2-weighted images; G, H: coronal T1-weighted images without and with gadolinium, respectively. Yellow arrows point to the dermal connection of the mass. Image courtesy: Radiology Department of Al Rasoul Al Aazam Hospital

A cranial and cervical magnetic resonance imaging (MRI) with gadolinium revealed a pedunculated sac-like mass resembling a myelomeningocele at the level of the second cervical vertebra. Details of the MRI are depicted in Figure 2.

**Figure 3:**
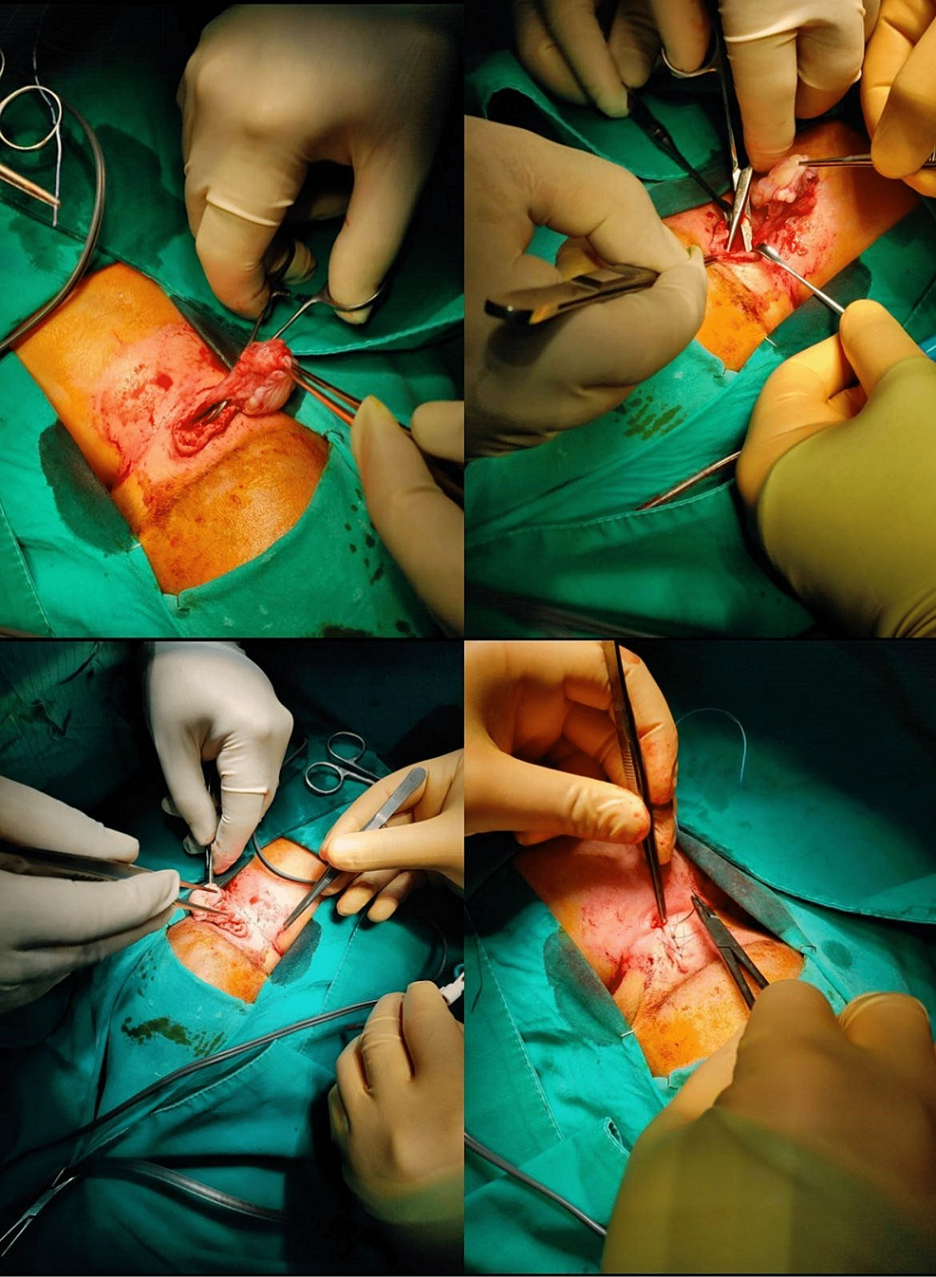
Intraoperative images showing the steps of surgery.

The patient was scheduled for surgery. Under general anesthesia and in the prone position, the mass was thoroughly opened to identify neural tissue. None were identified. The dural layer was closed in a water-tight fashion. The skin was sutured using 5-0 Vicryl and 5-0 nylon interrupted sutures (Figure 3).

Physical examination after surgery revealed normal upper and lower urinary and gastrointestinal activity. The specimen was sent to the pathology laboratory, which reported meningeal and skin samples devoid of any neural tissue.

## Discussion

Valeur et al. reported on the extreme rarity of cystic dysraphism of the cervical and upper thoracic spine and the better prognosis versus lumbosacral dysraphism given the lack of functional neurological tissue in the dysraphic sac [13]. Classification and nomenclature of cervical myelomeningoceles (cMMC) have evolved through the literature [14-19] (Table 1).

**Table 1:** Classification of cervical myelomeningoceles.

Publication details	Nomenclature
Maclone et al. [14]	cMMCs are closed spinal dysraphisms with a skin-covered posterior midline mass, narrow spina bifida, and a cerebrospinal fluid-filled cyst.
Steinbok et al. [15]	cMMCs are meningocele and myelocystocoeles.
Pang et al. [16]	Division according to internal features: fibro-neurovascular stalk and limited dorsal myelochisis.
Salomao et al., Habibi et al., and Rossi et al. [17,18,19]	cMMCs are fibro-neurovascular stalks and myelocystoceles.

Our case report adheres to the definition by Maclone et al. [14]. According to Steinbok et al., it can be termed a myelocystocele [15]. Patients with cMMCs have a better prognosis versus thoracolumbar or lumbosacral MMCs due to the rarity of spinal cord tethering and the slow ascension of the cervical cord during growth. This limits neurological damage due to cervical cord tethering [14,20].

Myelocystoceles are rare types of neural tube defects that involve the protrusion of the spinal cord and meninges through a defect in the vertebral column [15]. These defects are thought to result from incomplete neural tube closure during embryonic development. Myelocystoceles are typically classified based on their location and the type of tissue involved. Cervical myelocystoceles, in particular, are extremely uncommon and can present with various neurological problems and atrophies. Although the incidence of myelocystoceles is relatively low, the condition can have significant impacts on a patient's quality of life and can result in complications such as infections, tethered cord syndrome, and hydrocephalus. The management of myelocystoceles typically involves early diagnosis, multidisciplinary care, and surgical intervention to repair the affected vertebral column. The literature on myelocystoceles underscores the importance of increased awareness, surveillance, and research to improve the understanding, prevention, and treatment of this rare condition [17-19].

Cranial and cervical MRIs are indispensable in diagnosis and surgical planning [20]. Mainly, surgery is done to untether the cervical spinal cord and for cosmetic purposes. Achieving cosmesis without untethering the cord can be associated with neurological damage in the future and may warrant re-exploration [20]. Our patient showed no tethering on the MRI or during exploration. Cosmesis and extreme sterility were achieved.

## Conclusions

We have presented a rare case of cervical myelocystocele and reviewed the classifications of this condition within the literature. Early diagnosis and surgical intervention are crucial for the successful management of this rare condition, as it can lead to various neurological problems and complications such as tethered cord syndrome and hydrocephalus. Our case report highlights the importance of increased awareness and surveillance for myelomeningocele, particularly the rare cervical subtype. Overall, this report underscores the need for continued research to improve the prevention, diagnosis, and treatment of myelocystocele and other types of neural tube defects.
